# Intentions for catch-up HPV vaccination in Japan: an internet survey

**DOI:** 10.1007/s10147-023-02411-0

**Published:** 2023-09-30

**Authors:** Satoshi Nakagawa, Asami Yagi, Yutaka Ueda, Sayaka Ikeda, Mamoru Kakuda, Kosuke Hiramatsu, Ai Miyoshi, Toshihiro Kimura, Kei Hirai, Masayuki Sekine, Tomio Nakayama, Eiji Kobayashi, Etsuko Miyagi, Takayuki Enomoto, Tadashi Kimura

**Affiliations:** 1 Department of Obstetrics and Gynecology, Osaka University Graduate School of Medicine, 2-2, Yamadaoka, Suita, Osaka, 565-0871 Japan; 2 Clinical Psychology, Graduate School of Human Sciences, Osaka University, 1-2, Yamadaoka, Suita, Osaka, 565-0871 Japan; 3 Department of Obstetrics and Gynecology, Niigata University Graduate School of Medical and Dental Sciences, 1-757 Asahimachi-Dori, Chuo-Ku, Niigata, 951-8510 Japan; 4 Center for Public Health Sciences, National Cancer Center, 5-1-1, Tsukiji, Chuo-Ku, Tokyo, 104-0045 Japan; 5 Department of Obstetrics and Gynecology, Oita University Graduate School of Medicine, 1-1, Hasamamachiidaigaoka, Yufu, Oita 879-5593 Japan; 6 Department of Obstetrics and Gynecology, Yokohama City University Graduate School of Medicine, 3-9 Fukuura, Kanazawa-Ku, Yokohama, Kanagawa 236-0004 Japan

**Keywords:** Japan, HPV vaccine, Catch-up vaccination, Cervical cancer, Suspension of recommendation, Health communication

## Abstract

**Purpose:**

In Japan, Japan’s Ministry of Health, Labor, and Welfare decided to suspend govermental recommendation for HPV vaccination in FY 2013. The HPV vaccination rate for those born in FY 2000 or thereafter declined dramatically. In 2021, the “suspension of recommendation” ended. The catch-up vaccinations for the unvaccinated have been offered nationwide from FY 2022 to FY 2024. We aimed to quantify the vaccination intentions and characteristics of those young women now eligible for catch-up vaccination.

**Methods:**

In February of 2022, we conducted an internet survey targeted women who were born in 1997–2004 but who had not yet been HPV vaccinated.

**Results:**

We received 1,648 valid responses. 41.6% of the respondents wanted to uptake the catch-up HPV vaccination, 29.7% were undecided, and 28.7% did not want to be vaccinated. The intention to uptake catch-up HPV vaccination was associated with a good history of gynecological visits, intention to receive cervical cancer screening, sexual activity, degree of anxiety about cervical cancer, familiarity with problems associated with cervical cancer, experience with vaccination recommendations, and knowledge about cervical cancer (*p* < 0.05, respectively). In the vaccinated generation, the proportion of the group that did not want to be vaccinated was significantly higher (*p* < 0.05). In the vaccine-suspended generation, the proportion of the group that wanted to be vaccinated was significantly higher (*p* < 0.05).

**Conclusion:**

Our survey revealed that catch-up vaccination intentions differed depending on the vaccination environment. It is necessary for all organizations involved with HPV vaccination, such as government, medical institutions, and educational institutions, to make recommendations based on an understanding of the characteristics of the “vaccinated generation” and the “vaccine-suspended generation”.

## Background

Of the more than 200 types of human papillomavirus (HPV) known to infect humans, 14 have a high risk of causing cervical cancer. Two strains, HPV16 and HPV18, are associated with 70% of all cervical cancers worldwide, and roughly 60% in Japan [1–3]. HPV16 and 18 account for about 530,000 cervical cancer cases per year worldwide. HPV16/18 are also the most common strains in men’s HPV-related oropharyngeal cancer, accounting for 30,000 cases per year worldwide [4]. The main route of HPV transmission is sexual mucosal skin contact. Use of a condom will reduce HPV transmission to the cervix, but does not prevent it completely [5].

Over recent decades in Japan, the incidence of cervical cancer among young females has been increasing, and the peak incidence has been shifting to ever younger age groups [6]. With the introduction of the HPV vaccine as an effective countermeasure against the increasing cervical cancer and a public-funded vaccination program, there were great expectations for reducing these trends. Subsidies from the national and local governments for programs for bivalent-vaccination of girls aged 13–16 began in 2010. By 2013, both bivalent and quadrivalent HPV vaccines were being used routinely for vaccinating girls aged 12–16 as part of the National Immunization Program.

However, in June of 2013, due to media reports of possible post-vaccination adverse symptoms, Japan’s Ministry of Health, Labor, and Welfare (MHLW) decided to temporarily suspend the national immunization program’s recommendation for routine HPV vaccination [7]. As a result, the HPV vaccination rate for girls born in or after 2000 declined dramatically [8].

In December of 2015, the World Health Organization (WHO) Global Advisory Committee for Vaccine Safety issued a statement on HPV vaccine safety [9, 10], warning the Japanese government that “policy decisions based on weak evidence, leading to lack of use of safe and effective vaccines, can result in real harm.” (WHO) WHO’s warnings were not heeded, and Japan’s policy decisions based on weak evidence for adverse events led to a lack of use of the safe and effective vaccine [10, 11]. To counter the negative publicity, the vaccine was receiving, in April 2017, a nationwide epidemiological survey, led by a research team appointed by the MHLW, concluded that the putative “adverse symptoms” were not specific to females who were vaccinated [12–14].

In November of 2021, the MHLW’s “temporary suspension” of its active vaccination recommendation finally ended. The feared unwarranted ill effects of nearly 9 years of little to no HPV vaccinations nationwide have become the new reality.

The historical HPV vaccination rate, and thus the potential for infection, now varies greatly in Japan, depending on a girl or woman’s fiscal year (FY) of birth [15]. Sekine et al*.* reported the HPV infection rates among females aged 20–21 years old who were targeted for cervical cancer screening in the FY 2014–2020. They showed that infection rates increased for those born in 2000, the vaccine-suspended generation [10]. Furthermore, our group has reported that an increased rate of cervical cytological abnormalities is also occurring in the vaccine-suspended generation [11].

Recognizing that there is a tremendous added risk for cervical cancer for this “poorly vaccinated gap generation”, i.e., in those women who failed to get vaccinated during the 8 years when the government withheld its recommendation for doing so, Japan is now offering to cover the costs of their “catch-up vaccination”. The MHLW announced that these catch-up vaccinations would be administered from April of 2022 to March of 2025 for those born between 1997 and 2005 [16]. In addition, a 9-valent HPV vaccine (covering five additional high-risk types of HPV) is introduced into the routine vaccination program in April of 2023.

A previous study reported the pattern of HPV vaccine hesitancy among the catch-up generations in Japan [17], but the reasons for their intention to be vaccinated were not analyzed. Increasing the early catch-up vaccination rate is essential for cervical cancer control in Japan. In our study, more detailed information including intention for catch-up vaccination was collected and analyzed. We have conducted an internet survey to quantify the vaccination intentions and characteristics of those currently eligible for catch-up vaccination to determine where and how we need to apply assistance to that goal.

## Methods

### Study design

We conducted our cross-sectional study using internet survey in February of 2022. We targeted women born in 1997–2004 (1,648 in total, 206 for each of the FY of birth targeted for catch-up vaccinations) who had not yet been HPV vaccinated at the time of the survey.

### Setting

This internet survey was a cross-sectional study of monitor members selected from the panel of a market survey company, MACROMILL, INC., in Japan. Responders were selected based on the registered information of a group of ‘monitor members’ and their responses to a prescreening survey. Before our main HPV survey, 20,000 Japanese females, born in FY1997–2004 were invited by a web link which provided information about the study and pre-screened. The prescreening survey asked the respondents about their history of HPV vaccination. We collected data for area of residence, sex, age, household income, employment status, marital status, and the presence of children of the respondents. We conducted the prescreening survey from 15 to 16 February 2022. In this study, we used birth FY as allocation factors and selected respondents so that it would be equal to the residential area composition of the general population.

After the prescreening survey was conducted, we conducted the main survey, which asked about the following: what are your intentions to get the HPV catch-up vaccination, what are your reasons for this intention, are you living with mother, and what is your history of cervical cancer screening, intention to receive cervical cancer screening, history of gynecological visits, sexual activity, anxiety about cervical cancer, has someone close to you ever had cervical cancer, has your mother or family recommended HPV vaccination, what is your awareness of cervical cancer screening, and what is your knowledge about cervical cancer? We asked about her COVID vaccination status, and if not yet vaccinated, her intention to get the COVID vaccine. We also asked the inoculation status of the COVID-19 vaccine that attracted as much attention as the HPV vaccine in comparison. The collection of answers was stopped on the day the number of respondents per each birth FY became 206, with 1648 answers finally. We conducted the main survey from 17 to 20 February 2022.

### Participants

Participants were able to open the survey only after receiving the study information and after actively consenting to study participation. In Japan, the rate for compulsory education is as high as 99% [18]. The Internet usage for those in their teens and twenties is 98%, suggesting that there were no significant differences for the responders in their access to the Internet based on their social backgrounds [19]. Respondents who were inappropriate, such as those whose response time was extremely short, were excluded from the analysis.

### Variables/measurement

The questionnaire for the Internet survey was based on the interviews we conducted prior to our internet survey. The interview survey examined the barriers to catch-up vaccination among eight females of age for catch-up vaccination. The intention to catch-up HPV vaccination was asked as follows: “Would you like to be inoculated with the HPV vaccine on catch-up vaccination opportunities?” The respondents answered from the options of “want to be vaccinated”, “somewhat want to be vaccinated”, “unable to determine”, “do not want to be vaccinated so much”, and “do not want to be vaccinated”. In the analysis, the respondents were categorized into two groups: those who wanted to be vaccinated and those who were somewhat wanting to be vaccinated were categorized as the “want to be vaccinated” group, and those who were unable to determine, did not want to be vaccinated so much, and did not want to be vaccinated were categorized as “do not want to be vaccinated” group. The reasons for the intention to get the catch-up HPV vaccination were answered from the following options: very applicable, somewhat applicable, neither applicable nor not applicable, not applicable so much, and not applicable at all. In the analysis, the respondents were categorized into two groups: those who answered, “very applicable” or “somewhat applicable” were categorized as “applicable as a reason” group, and those who answered “neither applicable nor not applicable” or “not applicable so much” or “not applicable at all” were categorized as “not applicable as a reason” group.

HPV vaccination rates for girls in Japan significantly vary depending on year of birth. We defined two “generations of HPV vaccination” environments eligible for delayed catch-up vaccinations. The “vaccinated generation” was composed of women born in 1997–1999; the “vaccine-suspended generation” was made up of those born in 2000–2007 who initially came of HPV vaccination-eligibility age during the 9 years of recommendation suspension.

### Statistical methods

Statistical analysis was performed by the Chi-square test and by residual analysis. The statistical level of significance was set to be less than 5% bilaterally.

## Results

### Respondent characteristics

The backgrounds of our survey participants are shown in Table 1. Females born in 1997 and 1998 were more likely to be married and have children than women born in later birth years (*p* < 0.05, for both). More than 90% of women born in 1999 or after were unmarried, and especially those born in or after 2001 (*p* < 0.05). For those born in and after 2002 (20 or younger at the time of the survey), the proportion of those without children was significantly higher than those born before 2002 (*p* < 0.05). Household income was significantly higher among those born in 1997, with 29.6% having an annual household income of less than 4 million yen, 24.8% having an annual household income of between 4 and 8 million, and 28.6% having an annual household income of less than 4 million among those born in 1998 (*p* < 0.05, respectively). For work status, more than 60% of the respondents born in 1999 or later were students. The percentage of students was significantly higher for those born in 2001 and later than those born before 2001 (*p* < 0.05). The more recent the FY in which they were born, the higher the proportion of them still living with their mothers was.

**Table 1:** Respondent characteristics

	Birth fiscal year (FY) *n* (%)								Total
	1997	1998	1999	2000	2001	2002	2003	2004	
Number of births per FY	206	206	206	206	206	206	206	206	1,648
Marriage status									
Unmarried	155** (75.2)	180** (87.4)	194 (94.2)	195 (94.7)	202* (98.1)	205* (99.5)	206* (100.0)	205* (99.5)	1,542 (93.6)
Married	51* (24.8)	26* (12.6)	12 (5.8)	11 (5.3)	4** (1.9)	1** (0.5)	0** (0.0)	1** (0.5)	106 (6.4)
Presence of children									
Do not have	169** (82.0)	185** (89.8)	192 (93.2)	200 (97.1)	199 (96.6)	203* (98.5)	203* (98.5)	205* (99.5)	1,556 (94.4)
Have	37* (18.0)	21* (10.2)	14 (6.8)	6 (2.9)	7 (3.4)	3** (1.5)	3** (1.5)	1** (0.5)	92 (5.6)
Annual household income									
Less than four million yen	61* (29.6)	59* (28.6)	39 (18.9)	44 (21.4)	33 (16.0)	34 (16.5)	25** (12.1)	19** (9.2)	314 (19.1)
Four to eight million yen	51* (24.8)	34 (16.5)	27 (13.1)	24 (11.7)	16** (7.8)	16** (7.8)	14** (6.8)	29 (14.1)	211 (12.8)
Eight or more million yen	10 (4.9)	15 (7.3)	21 (10.2)	18 (8.7)	14 (6.8)	14 (6.8)	11 (5.3)	16 (7.8)	19 (7.2)
Do not know/no response	84** (40.8)	98** (47.6)	119 (57.8)	120 (58.3)	143* (69.4)	142* (68.9)	156* (75.7)	142* (68.9)	1,004 (60.9)
Work status									
Full-time worker	96* (46.6)	91* (44.2)	35 (17.0)	24 (11.7)	8** (3.9)	5** (2.4)	0** (0.0)	0** (0.0)	259 (15.7)
Part-time worker	39* (18.9)	40* (19.4)	23 (11.1)	17 (8.3)	16 (7.8)	11** (5.3)	3** (1.5)	1** (0.5)	150 (9.1)
Housewife	31* (15.0)	16* (7.8)	13 (6.3)	9 (4.4)	1** (0.5)	2** (1.0)	0** (0.0)	0** (0.0)	72 (4.4)
Student/other	20** (9.7)	34** (16.5)	124 (60.2)	145 (70.4)	173* (84.0)	181* (87.9)	202* (98.1)	203* (98.5)	1082 (65.7)
Unemployed	20* (9.7)	25* (12.1)	11 (5.3)	11 (5.3)	8 (3.9)	7 (3.4)	1** (0.5)	2** (1.0)	85 (5.2)
Living with mother									
Yes	89** (43.2)	110** (53.4)	146 (70.9)	138 (67.0)	145 (70.4)	146 (70.9)	198* (96.1)	201* (97.6)	1,173 (71.2)
No	117* (56.8)	96* (46.6)	60 (29.1)	68 (33.0)	61 (29.6)	60 (29.1)	8** (3.9)	5** (2.4)	475 (28.8)

^*^Significantly more

^**^Significantly less

*p* < 0.05, Chi-square test and residual analysis

### Factors that correlated with an intention to get the catch-up HPV vaccination

In regards to the HPV catch-up vaccination, 41.6% of the respondents wanted to receive it, 29.7% were undecided, and 28.7% did not want to be vaccinated (Table 2). A history of cervical cancer screening (in the past two years) was not associated with catch-up vaccination intentions (not significant, N.S.). Having had a recent previous gynecological visit was significantly more common in the group who wanted to be vaccinated (42.6%, *p* < 0.05). Regarding the intention to receive cervical cancer screening, in the group who wanted to be vaccinated, those who answered, “I intend to receive cervical cancer screening within the next two years” and “I would like to receive cervical cancer screening someday” were significantly higher than for the other two groups (21.6% and 52.2%, respectively) (*p* < 0.05, respectively). The group that did not want to be vaccinated had a significantly higher percentage of those having no intention to receive cervical cancer screening, 37.3% (*p* < 0.05). The proportion of respondents who reported having had sexual intercourse was significantly higher in the group that wanted to be vaccinated, 43.6% (*p* < 0.005).

**Table 2:** Factors that correlated with an intention to get the catch-up HPV vaccination

	Intention to HPV vaccine catch-up							
	Want to be vaccinated (*n* = 686, 41.6%)		Unable to determine vaccination intention (*n* = 490, 29.7%)		I do not want to be vaccinated (*n* = 472, 28.7%)		Total (*n* = 1,648)	
	*n*	%	*n*	%	*n*	%	*n*	%
History of cervical cancer screening in the past 2 years (Born in and before FY2001, 20 years old or older) N.S								
Yes	81	19.3	42	13.9	54	17.5	177	17.2
No	330	78.8	254	84.1	250	80.9	834	81.0
Do not know	8	1.9	6	2.00	5	1.6	19	1.8
History of gynecological visits								
Yes	292*	42.6	177	36.1	180	38.1	649	39.4
No	386	56.3	300	61.2	282	59.7	968	58.7
Do not know	8	1.2	13	2.7	10	2.1	31	1.9
History of gynecological visits (born in and before FY2001, 20 years old or older) N.S								
Yes	214	51.1	132	43.7	140	45.3	486	47.2
No	202	48.2	165	54.6	165	53.4	532	51.6
Do not know	3	0.7	5	1.7	4	1.3	12	1.2
Intention to receive cervical cancer screening								
I intend to receive cervical cancer screening within the next 2 years	148*	21.6	34**	6.9	50**	10.6	232	14.1
I would like to receive cervical cancer screening someday	358*	52.2	199	40.6	134**	28.4	691	41.9
I do not intend to receive cervical cancer screening	47**	6.9	79	16.1	176*	37.3	302	18.3
Do not know	133**	19.4	178*	36.3	112	23.7	423	25.7
Sexual activity								
I have had sexual intercourse	299*	43.6	165**	33.7	179	37.9	643	39.0
I have never had sexual intercourse	354	51.6	262	53.5	247	52.3	863	52.4
I do not want to answer	33**	4.8	63*	12.9	46	9.7	142	8.6
Degree of anxiety about cervical cancer								
Fairly or somewhat	537*	78.3	243**	49.6	210**	44.5	990	60.1
Not either	87**	12.7	152*	31.0	92	19.5	331	20.1
Not at all or not very much	62**	9.0	95	19.4	170*	36.0	327	19.8
Has someone close to the respondent had cervical cancer (Have you, your family, or anyone around you ever had cervical cancer?)								
Yes	29	4.2	20	4.1	25	5.3	74	4.5
No	526**	76.7	389	79.4	389*	82.4	1,304	79.1
Do not know	131*	19.1	81	16.5	58**	12.3	270	16.4
Has your mother or family recommended HPV vaccination								
Yes	151*	22.0	36**	7.3	39**	8.3	226	13.7
No	468**	68.2	394*	80.4	407*	86.2	1,269	77.0
Do not know	67	9.8	60*	12.2	26**	5.5	153	9.3
Awareness of cervical cancer screening								
Do you know that there is a test called cervical cancer screening?								
Yes	616*	89.8	392**	80.0	395	83.7	1,403	85.1
No	70**	10.2	98*	20.0	77	16.3	245	14.9
Knowledge about cervical cancer								
Cervical cancer may prevent pregnancy and childbirth								
Yes	612*	89.2	372**	75.9	366**	77.5	1,350	81.9
No	12**	1.7	12	2.4	27*	5.7	51	3.1
Do not know	62**	9.0	106*	21.6	79	16.7	247	15.0
Most cervical cancer is caused by HPV								
Yes	327*	47.7	186**	38.0	178**	37.7	691	41.9
No	65	9.5	37	7.6	55	11.7	157	9.5
Do not know	294**	42.9	267*	54.5	239	50.6	800	48.5

The percentage of respondents who reported having a higher level of anxiety about cervical cancer (fairly or somewhat worried) was 78.3% in the group who wanted to be vaccinated, which was significantly higher than in the other two groups (*p* < 0.05). In the group that did not want to be HPV vaccinated, the percentage of respondents who answered that they were not at all anxious, or were not very anxious, was 36.0%, significantly higher than for the other two groups (*p* < 0.05). Regarding the question, “Has someone close to you had cervical cancer?”, significantly more respondents in the group who do not want to be vaccinated answered “No” than in the other two groups (82.4%, *p* < 0.05).

Regarding the question, “Has your mother or your family recommended HPV vaccination?”, significantly more respondents in the group who wanted to be vaccinated answered “Yes” than in the other two groups (22.0%, *p* < 0.05). The percentage of those who answered “No” was significantly higher in the “Undecided” group (80.4%), and in the “I do not want to be vaccinated” group (86.2%), than in the “I want to be vaccinated” group (*p* < 0.05, respectively). Personal levels of awareness of cervical cancer screening and knowledge about cervical cancer were better in the “I want be vaccinated” group (89.8%, 89.2% and 47.7%, respectively), than in the “Undecided” group (20.0%, 21.6%, and 54.5%).

Table 3 shows the factors associated with intention of HPV catch-up vaccination. Those born in 1998 had the largest rate of the group that did not want to be vaccinated, and those born in 2001 had the largest rate of the group that wanted to be vaccinated. The group that wanted to be vaccinated had a higher rate of those who did not live with their mothers in both generations. The rate of those living with their mothers was significantly higher among the group who were undecided about vaccination in the vaccination generation. Among the vaccination generation, all four positive reasons were significantly more applicable to the group that intended to be vaccinated. The rate of negative reasons #1–3 not applicable as a reason for intention to vaccinate was significantly higher in the “Want to be vaccinated” group. The same result was observed for the vaccine-suspended generation.

**Table 3:** Factors associated with intention of HPV catch-up vaccination

	Intention to get catch-up HPV vaccination							
	Want to be vaccinated		Unable to determine vaccination intention		Do not want to be vaccinated		Total	
	*n*	%	*n*	%	*n*	%	*n*	%
Birth FY								
1997	89	13.0	50	10.2	67	14.2	206	12.5
1998	75	10.9	56	11.3	75*	15.9	206	12.5
1999	69**	10.1	78*	15.9	59	12.5	206	12.5
2000	84	12.2	62	12.7	60	12.7	206	12.5
2001	102*	14.9	56	11.5	48	10.2	206	12.5
2002	92	13.4	54	11.0	60	12.7	206	12.5
2003	89	13.0	72	14.7	45**	9.5	206	12.5
2004	86	12.5	62	12.7	58	12.3	206	12.5
Living with mother								
Vaccinated-generation (females born in 1997–1999)								
Yes	114**	51.1	119*	64.7	112	55.7	345	55.8
No	119*	48.9	65**	35.3	89	44.3	273	44.2
Vaccine-suspended generation (females born in 2000–2007)								
Yes	350**	77.3	257	84.0	221	81.6	828	80.4
No	103*	22.7	49	16.0	50	18.4	202	19.6
Vaccinated-generation (females born in 1997–1999)								
Positive reason								
1. Cervical cancer is a terrible disease								
Applicable as a reason	205*	88.0	87**	47.3	68**	33.8	360	58.2
Not applicable as a reason	28**	12.0	97*	52.7	133*	66.2	258	41.8
2. I am at risk of getting cervical cancer								
Applicable as a reason	196*	84.1	89	48.4	48**	23.9	333	53.9
Not applicable as a reason	37**	15.9	95	51.6	153*	76.1	285	46.1
3. If a vaccine can prevent cervical cancer, I want to prevent it								
Applicable as a reason	224*	96.1	101	54.9	33**	16.4	358	57.9
Not applicable as a reason	9**	3.9	83	45.1	168*	83.6	260	42.1
4. The HPV vaccine is free of charge								
Applicable as a reason	215*	92.3	81**	44.0	17**	8.5	313	50.7
Not applicable as a reason	18**	7.7	103*	56.0	184*	91.5	305	49.3
5. The HPV vaccine is recommended by the government								
Applicable as a reason	118*	50.6	31**	16.9	19**	9.5	168	27.2
Not applicable as a reason	115**	49.4	153*	83.1	182*	90.5	450	72.8
Negative reason								
1. I am afraid of adverse reactions								
Applicable as a reason	87**	37.3	142*	77.2	176*	87.6	405	65.5
Not applicable as a reason	146*	62.6	42**	22.8	25**	12.4	213	34.5
2. The HPV vaccine is unfamiliar to me								
Applicable as a reason	50**	21.5	104*	56.5	140*	69.7	294	47.6
Not applicable as a reason	183*	78.5	80**	43.5	61**	30.3	324	52.4
3. Getting the vaccine is a hassle								
Applicable as a reason	60**	25.8	107*	58.2	103*	51.2	270	43.7
Not applicable as a reason	173*	74.2	77**	41.8	98**	48.8	348	56.3
4. I intend to go for cervical cancer screening								
Applicable as a reason	96*	41.2	41**	22.3	50**	24.9	187	30.3
Not applicable as a reason	137**	58.8	143*	77.7	151*	75.1	431	69.7
Vaccine-suspended generation (Females born 2000–2007)								
Positive reason								
1. Cervical cancer is a terrible disease								
Applicable as a reason	388*	85.7	174**	56.9	79**	29.2	641	62.2
Not applicable as a reason	65**	14.3	132*	43.1	192*	70.8	389	37.8
2. I am at risk of getting cervical cancer								
Applicable as a reason	401*	88.5	164**	53.6	70**	25.8	635	61.7
Not applicable as a reason	52**	11.5	142*	46.4	201*	74.2	395	38.3
3. If a vaccine can prevent cervical cancer, I want to prevent it								
Applicable as a reason	443*	97.8	183**	59.8	44**	16.3	670	65.0
Not applicable as a reason	10**	2.2	123*	40.2	227*	83.7	360	35.0
4. The HPV vaccine is free of charge								
Applicable as a reason	415*	91.6	136**	44.4	20**	7.4	571	55.4
Not applicable as a reason	38**	8.4	170*	55.6	251*	92.6	459	44.6
5. The HPV vaccine is recommended by the government								
Applicable as a reason	247*	54.5	83**	27.1	21**	7.8	351	34.1
Not applicable as a reason	206**	45.5	223*	72.9	250*	92.2	679	65.9
Negative reason								
1. I am afraid of adverse reactions								
Applicable as a reason	176**	38.9	245*	80.1	243*	89.7	664	64.5
Not applicable as a reason	277*	61.1	61**	19.9	28**	10.3	366	35.5
2. The HPV vaccine is unfamiliar to me								
Applicable as a reason	106**	23.4	198*	64.7	212*	78.2	516	50.1
Not applicable as a reason	347*	76.6	108**	35.3	59**	21.8	514	49.9
3. Getting the vaccine is a hassle								
Applicable as a reason	84**	18.5	133*	43.5	123*	45.4	340	33.0
Not applicable as a reason	369*	81.5	173**	56.5	148**	54.6	690	67.0
4. I intend to go for cervical cancer screening W								
Applicable as a reason	172*	38.0	54**	17.7	60**	22.1	286	27.8
Not applicable as a reason	281**	62.0	252*	82.3	211*	77.9	744	72.2

^*^Significantly more

^**^Significantly less

*p* < 0.05, Chi-square test and residual analysis, bilaterally

### Reasons associated with intending or not intending to get the HPV catch-up vaccination

The respondent’s reasons for their intention to receive or not receive the catch-up HPV vaccination are shown in Table 4. For those who wanted to be vaccinated, the positive reasons 1–4 were chosen significantly more often than other reasons for the intention to be vaccinated (*p* < 0.05, respectively). “HPV vaccine is recommended by the government” was not selected as a reason for intention to be vaccinated significantly more than other positive reasons (*p* < 0.05).

**Table 4:** Reasons for intention to inoculate HPV vaccine catch-up vaccination

	Applicable as a reason *n* (%)	Not applicable as a reason *n* (%)
Want to be vaccinate (*n* = 686)		
Positive reason		
1. Cervical cancer is a terrible disease	593 (86.4)*	93 (13.6)**
2. I am at risk of getting cervical cancer	597 (87.0) *	89 (13.0)**
3. If a vaccine can prevent cervical cancer, I want to prevent it	667 (97.2)*	19 (2.8)**
4. The HPV vaccine is free of charge	630 (91.8)*	56 (8.2)**
5. The HPV vaccine is recommended by the government	365 (53.2)**	321 (46.8)*
Negative reason		
1. I am afraid of adverse reactions	263 (38.4)*	423 (61.6)**
2. The HPV vaccine is unfamiliar to me	156 (22.7)**	530 (77.3)*
3. Getting the vaccine is a hassle	144 (21.0)**	542 (79.0)*
4. I intend to go for cervical cancer screening	268 (38.5)*	428 (61.5)**
Unable to determine vaccination intention (*n* = 490)		
Positive reason		
1. Cervical cancer is a terrible disease	261 (53.3)*	229 (46.7)**
2. I am at risk of getting cervical cancer	253 (51.6)*	237 (48.4)**
3. If a vaccine can prevent cervical cancer, I want to prevent it	284 (58.0)*	206 (42.0)**
4. The HPV vaccine is free of charge	217 (44.3)	273 (55.7)
5. The HPV vaccine is recommended by the government	114 (23.3)**	376 (76.7)*
Negative reason		
1. I am afraid of adverse reactions	387 (79.0)*	103 (21.0)**
2. The HPV vaccine is unfamiliar to me	302 (61.6)*	188 (38.4)**
3. Getting the vaccine is a hassle	240 (49.0)	250 (51.0)
4. I intend to go for cervical cancer screening	95 (19.4)**	395 (80.6)*
Do not want to be vaccinated (*n* = 472)		
Positive reason		
1. Cervical cancer is a terrible disease	147 (31.1)*	325 (68.9)**
2. I am at risk of getting cervical cancer	118 (25.0)*	354 (75.0)**
3. If a vaccine can prevent cervical cancer, I want to prevent it	77 (16.3)**	395 (83.7)*
4. The HPV vaccine is free of charge	37 (7.8)**	435 (92.2)*
5. The HPV vaccine is recommended by the government	110 (23.3)	362 (76.7)
Negative reason		
1. I am afraid of adverse reactions	419 (88.8)*	53 (11.2)**
2. The HPV vaccine is unfamiliar to me	352 (74.6)*	120 (25.4)**
3. Getting the vaccine is a hassle	226 (47.9)**	246 (52.1)*
4. I intend to go for cervical cancer screening	110 (23.3)**	362 (76.7)*

^*^Significantly more

^**^Significantly less

*p* < 0.05, Chi-square test and residual analysis, bilaterally

For the “Unable to determine vaccination intention” group, the positive reasons 1, positive reason 3, negative reason 1, and negative reason 2 were selected as significantly more the reasons for vaccination intention than the other reasons (*p* < 0.05, respectively). For the “Do not want to be vaccinated” group, the negative reason 1 and negative reason 2 were selected as significantly more the reasons for vaccination intention than the other reasons (*p* < 0.05, respectively).

### Comparison by HPV generation of the HPV vaccine catch-up intention, COVID vaccination status, and intention to get a COVID vaccination

In the defined “vaccinated generation”, the proportion that wanted to get the catch-up vaccination was significantly lower, and the proportion of the group that clearly did not want to be vaccinated was accordingly significantly higher (*p* < 0.05, respectively) (Table 5). In the vaccine-suspended generation, the proportion of the group that wanted to be vaccinated was significantly higher and the proportion of the group that did not want to be vaccinated was significantly lower (*p* < 0.05, respectively).

**Table 5:** Comparison by HPV generation of the HPV vaccine catch-up intention, COVID vaccination status, and intention to get a COVID vaccination

	Intention get catch-up HPV vaccination						COVID vaccination status and intention to get the COVID vaccine						Total
	Want to be vaccinated		Unable to determine vaccination intention		Do not want to be vaccinated		Already vaccinated at least once		Intend to be vaccinated in the future		Do not intend to be vaccinated		
	n	%	n	%	n	%	n	%	n	%	n	%	n
Vaccinated-generation (Females born 1997–1999)	233**	37.7	184	29.8	201*	32.5	409**	66.2	50**	8.1	159*	25.7	618
Vaccine-suspended generation (Females born 2000–2007)	453*	44.0	306	29.7	271**	26.3	733*	71.2	123*	11.9	174**	16.9	1,030
Total	686	41.6	490	29.7	472	28.7	1,142	69.3	173	10.5	333	20.2	1,648

^*^Significantly more

^**^Significantly less

*p* < 0.05, chi-square test and residual analysis, bilaterally

In the “HPV vaccinated generation”, the proportion of the group that self-reported as having been “Already been vaccinated for COVID at least once” or “I intend to be vaccinated for COVID in the future” was significantly lower, and the proportion of the group that responded “I do not intend to be COVID vaccinated” was significantly higher (*p* < 0.05, respectively). In the vaccine-suspended generation, the proportion of the group that had “Already been vaccinated for COVID at least once” or “I intend to be vaccinated for COVID in the future” was significantly higher, and the proportion of the group that responded “I do not intend to be COVID vaccinated” was significantly lower (*p*<0.05, respectively).

### Comparison between catch-up HPV vaccination intention, COVID vaccination status, and COVID vaccination intention (Table 6)

**Table 6:** Comparison between catch-up HPV vaccination intention, COVID vaccination status, and COVID vaccination intention

	Intention to get catch-up HPV vaccination							
	Want to be vaccinated		Unable to determine vaccination intention		Do not want to be vaccinated		Total	
	n	%	n	%	n	%	n	%
COVID vaccination status and intention to get the COVID vaccine								
Already vaccinated at least once	537*	47.0	329	28.8	276**	24.2	1,142	100.0
Intend to be vaccinated in the future	104*	60.1	58	33.5	11**	6.4	173	100.0
Do not intend to be vaccinated	45**	13.5	103	30.9	185*	55.6	333	100.0
Total	686	41.6	490	29.7	472	28.6	1,648	100.0

^*^Significantly more

^**^Significantly less

*p* < 0.05, Chi-square test and residual analysis, bilaterally

In the group that wanted to get the catch-up HPV vaccination, the proportion that responded “Already been vaccinated for COVID at least once” or “I intend to be vaccinated for COVID in the future” was significantly higher, and the proportion of the group that responded “I do not intend to be vaccinated for COVID” was significantly lower (*p* < 0.05, respectively). In the group that would refuse the catch-up HPV vaccination, the proportion that answered, “I have already been vaccinated for COVID at least once” and “I intend to be vaccinated for COVID in the future” was significantly lower, and the proportion of the group that “Do not intend to be vaccinated” was significantly higher (*p* < 0.05, respectively).

## Discussion

We conducted an internet survey of young women in Japan who were at that time beyond the normal grade school age of eligibility to receive a publicly subsidized HPV vaccination, but were now qualified for a government-recommended catch-up vaccination program meant to compensate them for 8 years of lapsed HPV vaccinations. We found that these young women’s intention to receive the catch-up vaccination was heavily associated with their prior history of gynecological visits, sexual activity, degree of anxiety about cervical cancer, intention to receive cervical cancer screening, familiarity with problems related to cervical cancer, whether they had received a recommendation for the vaccination from family, and their knowledge regarding cervical cancer.

The survey found that, compared to the “I want to be HPV vaccinated” group, the “Unable to determine my vaccination intention” and “I do not want to be vaccinated” groups were about twice as likely to answer they were afraid of the vaccine’s potential adverse side effects. The group who wanted to be vaccinated had a better history of gynecological visits, had a higher intention to receive cervical cancer screening, and were more knowledgeable about the cervical cancer. Furthermore, if they already had sexual intercourse, they were more anxious about their risks of cervical cancer. Therefore, we think they were more cognitive that “infection prevention meant cervical cancer prevention”, and thus they had a higher intention to be vaccinated, even though they too were afraid of the reported potential for adverse vaccination events.

Unexpectedly, there was a significantly lower proportion of respondents who wanted the catch-up HPV vaccination in the “vaccinated generation” compared to the “vaccine-suspended generation”. We are guessing that the reason for this surprising result is that the survey respondents from the “vaccinated generation” who were now refusing the catch-up vaccination were actually the small number of originally unvaccinated girls who now, years later, are being consistent with their previous choice not to be vaccinated.

This negative outcome is consistent with known human behavior that, once a stance has been chosen, it seldom changes without drastic reasons to do so. We assume that the vaccination rate among the “unvaccinated generation” has a higher possibility of benefiting from now providing them with targeted accurate information. This hope is because girls were born in FY2000 and later were at one time motivated to be vaccinated but then refrained, primarily in response to spurious reports of vaccination adverse side effects and the withdrawal of MHLW’s recommendation for the vaccination.

Knowing which women still need to be vaccinated depends on accessing their medical vaccination records. In Japan, under the Immunization Law, it is the local governments that are responsible for maintaining vaccination history data. However, the minimal data retention period, set at 5 years, has passed or will soon pass for these women, and some local governments have already begun discarding the very data we desperately need [20].

We found that the respondent’s COVID vaccination status and their intention to seek a COVID vaccination were well associated with their intention to also receive the HPV catch-up vaccination. The group who most wanted to be vaccinated was also more receptive to the COVID vaccine. This suggests to us that it might be more effective to match the specific catch-up HPV vaccination recommendation message to the number of COVID vaccination shots that the targeted woman has had.

In addition, in this survey, interesting findings were obtained regarding cervical cancer screening, which is important as secondary prevention of cervical cancer. The rate of respondents who had received a cervical cancer screening was less than half of the rate of those who answered that they had visited a gynecologist before. It was suggested that although younger females had opportunities to visit a gynecologist, these opportunities have not been connected to cervical cancer screening [21].

Like the results of a previous study that “parental intention to vaccinate and having a friend who had engaged in sexual intercourse were significant independent predictors of adolescents’ intent to accept STI vaccination” [22], our survey showed the group with high receptivity to the catch-up vaccination had their own sexual experience and their families had recommended vaccination. Several other reports have indicated that parental acceptance is important for immunization [23, 24]. In addition, a recommendation from a health care provider was shown to be associated with vaccine acceptability among parents [25–27]. In our survey, as mentioned earlier, the result was “the group who wanted to be vaccinated had a better history of gynecological visits, had a higher intention to receive cervical cancer screening, and were more knowledgeable about the cervical cancer”, which suggests that they may have obtained knowledge about screening and cervical cancer at the clinic. On the hand, a recommendation by a health care provider may be not enough to motivate mothers to vaccinate their daughters [28]. In the survey by Khodadadi et al., 35.3% of 317 respondents hesitated to vaccinate their daughters even when recommended by a doctor. They mentioned as follows, “Further efforts should focus on increasing awareness regarding HPV and cervical cancer, heightening perceived risk of HPV infection among daughters and boosting self-efficacy to get their children vaccinated against HPV”. We believe these efforts are important to increase the low vaccination rate in Japan as well. Because in our study, even in the high vaccination intention group, only 22% of the family members recommended the catch-up vaccination, and in the low vaccination intention group, less than 10% of the family members recommended the catch-up vaccination. More parents in Japan hesitate to vaccinate their children than in other countries [29].

Vaccine hesitancy is complex and context specific [30]. It is influenced by a wide variety of factors, including time, place, vaccine type, individual characteristics, social environment, and culture. Like our report this time, Machida et al. also reported that the HPV vaccine was highly accepted among those who received the COVID-19 vaccine. It would be effective to strengthen recommendations for this group first [17].

One of the limitations of our study is that we stratified the respondents into groups based on the birth FY, so the average age of the respondents was unavoidably significantly different in each group. In addition, we compared between the vaccinated generation and the vaccine-suspended generation, but it might be a comparison by different age groups. However, the social impact of the Japanese government’s decision has been significant, drastically reducing HPV vaccination rates. This impact is considered stronger than the impact of age on vaccination intentions. Second, the respondents were monitor members already enrolled in the consumer panel. Because the respondents have chosen to respond to this survey, some kind of sample selection bias may exist. In addition, generalizing the present results obtained from the internet survey may be problematic in view of the correspondence between the survey methodology and the respondent populations.

In conclusion, we have quantified the vaccination intentions and characteristics of the population of young women currently eligible for targeted catch-up HPV vaccination. The ages of those who were eligible for catch-up immunization in the year the catch-up immunization began (FY2022) were 17–25. In Japan, the current rate for female sexual intercourse experience at age 17 is about 25%. However, this rate increases significantly, to about 80%, by age 25. (JFPA) We need to encourage targeted females to start catch-up vaccinations as early as possible. It is necessary for all organizations involved with females eligible for HPV vaccination and their parents, such as government, medical institutions, and educational institutions, to make recommendations based on an understanding of the characteristics of the “vaccinated generation” and the “vaccine-suspended generation”.

## Abbreviations

HPV: Human papillomavirus
FY: Fiscal year
WHO: World Health Organization
MHLW: Ministry of health, labor, and welfare
